# Phenotypic Plasticity of Photochemical Traits and Antioxidant Responsiveness Confer Photosynthetic Resilience in Peanut (*Arachis hypogaea* L.) Under Phosphorus Deficiency: The Pivotal Role of Cyclic Electron Flow

**DOI:** 10.3390/antiox15081002

**Published:** 2026-08-12

**Authors:** Zhiyu Sun, Mingzhu Ma, Huan Liu, Md. Nasir Hossain Sani, Yifei Liu, Jean Wan Hong Yong

**Affiliations:** 1 China-Australia Joint Laboratory for Plant Nutrition and Germplasm Resources Innovation, College of Land and Environment, National Engineering Research Center for Efficient Utilization of Soil and Fertilizer Resources, Northeast China Plant Nutrition and Fertilization Scientific Observation and Research Center for Ministry of Agriculture and Rural Affairs, Key Laboratory of Protected Horticulture of Education Ministry and Liaoning Province, Shenyang Agricultural University, Shenyang 110866, China; sunzy@stu.syau.edu.cn (Z.S.); 2023200144@stu.syau.edu.cn (M.M.);; 2 Department of Nutritional Crop Physiology, Institute of Crop Science, University of Hohenheim, 70593 Stuttgart, Germany; 3 Department of Biosystems and Technology, Swedish University of Agricultural Sciences, 23456 Alnarp, Sweden; md.sani@slu.se; 4 Institute of Agriculture, The University of Western Australia, Perth, WA 6009, Australia; 5 School of Biological Sciences, The University of Western Australia, Perth, WA 6009, Australia; 6 School of Agriculture and Environment, The University of Western Australia, Perth, WA 6009, Australia; 7 Brightlands Future of Farming Institute, Faculty of Science and Engineering, Maastricht University, 5928 SX Venlo, The Netherlands

**Keywords:** cyclic electron flow, non-photochemical quenching, trans-thylakoid proton gradient, phosphorus deficiency, phenotypic plasticity, homeostasis

## Abstract

Phosphorus (P) deficiency is a major factor governing peanut (*Arachis hypogaea* L.) productivity, and the physiological mechanisms by which different genotypes (with contrasting photosynthetic capacities) coordinate carbon assimilation and photoprotection remain elusive. This study elucidated the strategic divergence among different peanut genotypes in their foliar photosystems to perform physiological homeostasis under low-phosphorus (LP) conditions. Based on a peanut mini-core collection, six representative accessions with contrasting photosynthetic capacities were selected and categorized into high- and low-photosynthetic functional groups. We integrated leaf gas exchange, chlorophyll fluorescence, the trans-thylakoid proton gradient (ΔpH), and antioxidant enzyme assays to evaluate their adaptive responses to low-P stress relative to the high-P (HP) control. Our results demonstrated that LP stress induced widespread photosynthetic inhibition across all accessions; this suppression was primarily driven by non-stomatal limitations. Under LP stress, high-Pn accessions exhibited superior cyclic electron flow (CEF) plasticity synergized with highly plastic guaiacol peroxidase (POD) activity, suppressing the leaf-level ROS burst and maintaining a substantial ΔpH for ATP synthesis and PSI stability. Conversely, low-Pn accessions suffered from severe oxidative overload and relied heavily on passive thermal dissipation, characterized by elevated non-photochemical quenching (NPQ) values and restricted CEF engagement. Principal component analysis (PCA) confirmed that while baseline biochemical impairments were universal, the capacity to dynamically modulate this ΔpH-dependent regulatory network—which integrates CEF, cytochrome b6f photosynthetic control, and antenna-level NPQ—served as the decisive determinant underlying genotypic variations in photosystem resilience under P deficiency. This study demonstrated that peanut genotypes deploy divergent, ΔpH-centered strategies to balance light energy distribution under P-limited conditions. These findings provide a novel and plausible mechanistic framework for selecting and breeding P-efficient peanut cultivars in poor soils with enhanced photosystem resilience.

## 1. Introduction

Phosphorus (P) is an essential macronutrient required for crop growth and development, playing an indispensable role in critical biological processes, including forming the structural formation of the nucleic acid backbone, signal transduction via phosphorylation, and transformation through adenosine triphosphate (ATP) [1,2]. Although the total P concentration in agricultural soils is often high, orthophosphate (P_i_, primarily as H_2_PO_4_^−^ and HPO_4_^2−^) is readily immobilized by soil minerals and exhibits poor mobility, resulting in severely restricted bioavailability for root uptake [3,4]. Consequently, P deficiency represents a primary constraint limiting global crop production. To sustain high yields in intensive agricultural systems, substantial amounts of phosphate fertilizers are routinely applied [5]. However, due to the high P-fixing capacity of soils, the seasonal utilization efficiency of these fertilizers remains extremely low (10–15%) [6]. The unutilized P accumulates in the soil profile or escapes into aquatic ecosystems via precipitation and surface runoff, triggering severe environmental degradation such as eutrophication [5]. Furthermore, commercial phosphate fertilizers are predominantly derived from non-renewable phosphate rock, a finite resource that is rapidly depleting [4,7]. Therefore, enhancing crop tolerance to low-P conditions and optimizing biological P-use efficiency represent a highly promising and sustainable agricultural strategy to mitigate fertilizer reliance in modern agriculture.

As a globally important oilseed crop, peanut *(Arachis hypogaea* L.) is frequently cultivated in marginal soils, making its production highly susceptible to P deficiency [8,9,10,11,12,13]. Among the various physiological processes impaired by low-P stress, the inhibition of leaf photosynthesis is the primary and most detrimental consequence limiting peanut productivity [10,14]. P deficiency inherently stunts plant growth, leading to a reduced demand for photoassimilates [10,15]. This diminished sink strength triggers the accumulation of sugars in leaves, which subsequently exerts feedback inhibition on the dark reactions of photosynthesis [10,15]. Concurrently, low-P stress imposes substrate limitations on ATP synthase, leading to an excessive accumulation of protons (H^+^) within the thylakoid lumen [16]. The resultant lumen acidification restricts the linear electron transport (LET) rate via the cytochrome b_6_f complex, thereby reducing the transfer of excess electrons from photosystem II (PSII) to photosystem I (PSI)—a vital regulatory phenomenon termed “photosynthetic control” [16]. Additionally, photosynthetic control serves to maintain the stoichiometric balance between ATP/NADPH photoproduction and its metabolic demand in the Calvin–Benson cycle [17]. Consequently, this photosynthetic control serves as an intrinsic photoprotective brake that mitigates the over-reduction in upstream electron carriers, effectively alleviating low-P stress-induced reactive oxygen species (ROS) bursts [18]. Excess ROS can disrupt photosynthetic machinery and compromise the structural integrity of thylakoid membranes [19]. To mitigate this photo-oxidative stress, plants mobilize robust enzymatic and non-enzymatic antioxidant defense systems to neutralize excessive ROS accumulation [18,20]. Therefore, elucidating how distinct peanut genotypes coordinate energy distribution and deploy downstream photoprotective strategies under LP stress is critical for mitigating oxidative impairment and sustaining agricultural productivity.

Plants have evolved sophisticated photoprotective strategies to mitigate potential photodamage under adverse environmental conditions [20]. Specifically, CEF around PSI effectively mitigates the excitation pressure derived from PSII, thereby safeguarding both reaction centers from photoinhibition [21,22]. In addition to driving ATP synthesis, CEF promotes energy-dependent quenching (qE) by elevating the thylakoid ΔpH [23,24]. Complementarily, non-photochemical quenching (NPQ) facilitates the safe dissipation of surplus excitation energy as heat, minimizing the risk of electron congestion within the thylakoid membrane [20,25]. Mechanistically, CEF pumps protons into the thylakoid lumen without the net accumulation of reducing equivalents, generating a robust trans-thylakoid proton gradient (ΔpH) that serves as the indispensable trigger for energy-dependent NPQ activation [26,27]. Consequently, ΔpH-dependent “photosynthetic control” represents a critical regulatory pivot for preserving PSI integrity under low-P stress [14,16]. Although the baseline growth characteristics and general physiological responses of peanut to phosphorus deficiency have been extensively documented [10,14,18,28,29], the inter-genotypic variation in the adaptive plasticity of photochemical traits—and how this plasticity coordinates with Pn stability—remains poorly understood.

In our previous studies, we systematically evaluated the physiological responses of peanut to varying P supplies, establishing critical concentration thresholds ranging from optimal to deficient levels, and demonstrated that even mild P deficiency can trigger severe photoinhibition [28]. While our previous studies showed that exogenous regulators (e.g., GABA, Ca, Mg) can mitigate low-P-induced photoinhibition [10,14,18,29], the intrinsic, genotype-dependent photoprotective mechanisms remain poorly understood. Although previous studies have documented root morphological plasticity and nutrient mobilization capacities under LP stress in peanut [30,31], research regarding genotypic variations in low-P resistance across diverse peanut cultivars remains surprisingly limited. Moreover, these investigations predominantly focused on below-ground traits or exogenous agronomic interventions. Consequently, the intrinsic, endogenous physiological mechanisms—particularly how native LP-tolerant genotypes orchestrate photoprotection to counteract photoinhibition—remain largely elusive.

Therefore, the present study selected six representative peanut accessions exhibiting contrasting photosynthetic rates under low-P. By employing concurrent gas exchange and simultaneous PSI and PSII dual-wavelength chlorophyll fluorescence measurements, we systematically investigated the dynamic coordination of energy partitioning between both photosystems. We hypothesized that: (i) peanut accessions with contrasting Pn deploy divergent photoprotective strategies, particularly regarding their flexible capacities to upregulate biophysical energy dissipation (NPQ and CEF) and coordinate downstream antioxidant defense (CAT and POD) to prevent oxidative stress chain propagation; and (ii) the robust activation of CEF around PSI serving as a pivotal endogenous driver, synergistically coupled with highly plastic localized antioxidant scavenging, to shield the photosynthetic apparatus against LP-induced photoinhibition and stabilize carbon assimilation.

## 2. Materials and Methods

### 2.1. Plant Sampling and Measurements

This study was conducted out at Shenyang Agricultural University (Shenyang, China) during the 2023 growing season. Six peanut accessions characterized by distinct photosynthetic capacities were selected from a mini-core collection and categorized into two functional groups: a high-photosynthetic-capacity (H) group and a low-photosynthetic-capacity (L) group. Two phosphorus (P) supply regimes were established, specifically a low-P (LP) stress treatment (15 mg P kg^−1^) and a high-P (HP) control treatment (54 mg P kg^−1^). Plants were cultivated in plastic pots with a single plant per pot. For each peanut accession under each P level, at least six pots (plants) were grown simultaneously under identical conditions. Subsequently, three healthy and uniform plants per treatment were selected as biological replicates (*n* = 3) for physiological and biochemical measurements.

A composite substrate consisting of Liaoning brown soil and washed, sun-dried river sand in a 1:1 (*v*/*v*) ratio was used in this study. The physicochemical properties of the baseline substrate were as follows: available nitrogen (N), 85.2 mg N kg^−1^; available potassium (K), 142 mg K kg^−1^; Olsen-P, 15.03 mg P kg^−1^; exchangeable calcium (Ca), 0.92 g Ca kg^−1^; exchangeable magnesium (Mg), 174 mg Mg kg^−1^; organic matter, 13.4 g kg^−1^; and pH 6.82 (measured in 10 mmol L^−1^ CaCl_2_, 1:2.5, *w*/*v*).

Based on our previous screening [28], a phosphorus (P) concentration of 15 mg P kg^−1^ was selected as the sub-optimal level (hereafter referred to as low-P, LP) for peanut cultivation. Prior to sowing, fertilizers were thoroughly homogenized with the soil. For the HP treatment, 14.4 g superphosphate was applied per pot. To decouple the nutritional effects of calcium from phosphorus, the LP pots were supplemented with 12.4 g CaSO_4_·2H_2_O per pot, thereby strictly balancing the total calcium input across both P regimes. A mobile rain shelter was deployed to exclude natural precipitation. Irrigation, weeding, and pest management were performed manually in accordance with regional conventional agronomic practices.

### 2.2. Leaf Gas Exchange Measurements and Real-Time Accession Grouping

At 36 days after planting (DAPs), the net photosynthetic gas exchange parameters were monitored on the third youngest fully expanded leaf on the main stem with an open gas exchange system (GFS 3000; Heinz Walz, Effeltrich, Germany). The measurements were taken at a photosynthetic photon flux density (PPFD) of 1000 μmol photons m^−2^ s^−1^ and a constant airflow rate of 750 μmol s^−1^. The concentration of CO_2_, leaf cuvette relative humidity, and temperature were set to 400 μmol CO_2_ mol^−1^, 60%, and 25 °C, respectively. Crucially, this real-time photosynthetic profiling across the mini-core collection served as the direct empirical basis for the immediate screening and functional segregation of the contrasting high-Pn (H) and low-Pn (L) groups for subsequent downstream analyses.

### 2.3. Chlorophyll Fluorescence and P_700_ Redox State Measurements

At 39 DAPs (three days after the initial Pn-based grouping), the chlorophyll fluorescence parameters and P700 redox states were concurrently monitored using a Dual-PAM-100 system (Heinz Walz, Effeltrich, Germany). These measurements were performed on the third youngest fully expanded leaf on the main stem previously utilized for the gas exchange analysis, adhering to established protocols optimized for peanut [9,10]. The absolute rate of cyclic electron flow (CEF) was calculated as the difference between the quantum yields or electron transport rates of photosystem I (PSI) and photosystem II (PSII) according to the following equation: CEF =  ETR(I) − ETR(II) [8]. The trans-thylakoid proton gradient (ΔpH) was determined via electrochromic shift (ECS) measurements utilizing a Dual-PAM-100 system equipped with a P515/535 emitter–detector module (Heinz Walz, Effeltrich, Germany). The quantification and partitioning of ΔpH from the dark relaxation kinetics of the P515 signal were calculated in accordance with the established protocols described by Sun, et al. [18].

### 2.4. Oxidative Stress Assays and Antioxidant Enzyme Activities

At 40 DAPs, the concentrations of hydrogen peroxide (H_2_O_2_) and malondialdehyde (MDA), as well as the activities of catalase (CAT) and peroxidase (POD), were determined in peanut leaves. The endogenous hydrogen peroxide H_2_O_2_ concentration was colorimetrically determined via the titanium tetrachloride precipitation method at 415 nm [32]. The MDA concentration was determined using the thiobarbituric acid (TBA) reaction, calculated from absorbances at 450, 532, and 600 nm [33]. For enzymatic assays, CAT activity was evaluated based on the linear decrease in H_2_O_2_ absorbance at 240 nm [34]; meanwhile, peroxidase (POD) activity was colorimetrically determined at 470 nm using the guaiacol method [35].

### 2.5. Phenotypic Plasticity Assessment

To quantify the phenotypic plasticity of photochemical traits under low-P stress, the LP-response effect was determined as the effect size (%) using the following formula: effect size (%) = (V_LP_ − V_HP_)/V_HP_ × 100_._ where V_LP_ and V_HP_ represent the mean values of a specific trait under low-P and high-P conditions, respectively [36].

### 2.6. Statistical Analyses

The experiment was arranged in a randomized complete block design. Data normality and homogeneity of variance were verified prior to analysis. For absolute functional traits, a two-way analysis of variance (ANOVA) was performed to evaluate the main effects of phosphorus (P) treatment, accession group, and their interaction (Table S1). For relative response effect sizes, data were analyzed using a one-way ANOVA. Post hoc comparisons were conducted using Fisher’s least significant difference (LSD) test in Origin 2025. All figures were generated using GraphPad Prism 9.5 software, while principal component analysis (PCA) was conducted in Origin 2025. Data are presented as means ± standard error (SE) and independent biological replicates (n = 3).

## 3. Results

### 3.1. Photosynthetic Performance and Accession Grouping

To evaluate the adaptive capacity of selected peanut accessions under LP stress, key photosynthetic traits were quantified. A multivariate cluster analysis (integrated with principal component analysis, PCA) based on photosynthetic parameters was conducted to rigorously validate genotype segregation. This statistical modeling confirmed that the six accessions naturally clustered into two distinct groups: a high-Pn (H) group and a low-Pn (L) group (Table 1; as supported by subsequent PCA). Significant variation in Pn was observed across the investigated accessions, with values ranging from 15.54 to 22.83 μmol CO_2_ m^−2^ s^−1^. Notably, the mean Pn of the H group (21.88 μmol CO_2_ m^−2^ s^−1^) was significantly higher than that of the L group (16.40 μmol CO_2_ m^−2^ s^−1^), demonstrating a clear statistical demarcation between the two groups (Table 1). Within the H group, accessions ICG7969, ICG9418, and ICG13603 maintained superior photosynthetic capacity, with ICG7969 achieving the highest Pn value of 22.83 μmol CO_2_ m^−2^ s^−1^. Conversely, the L group was characterized by significantly lower photosynthetic capacities; in particular, ICG1101 and ICG1448 displayed the most pronounced inhibition, with Pn values of only 15.54 and 15.95 μmol CO_2_ m^−2^ s^−1^, respectively (Table 1). The statistical analysis (a–f) revealed clear segregation not only between the two major groups but also among individual accessions within each group.

### 3.2. Photosynthetic Gas Exchange

**Figure 1:**
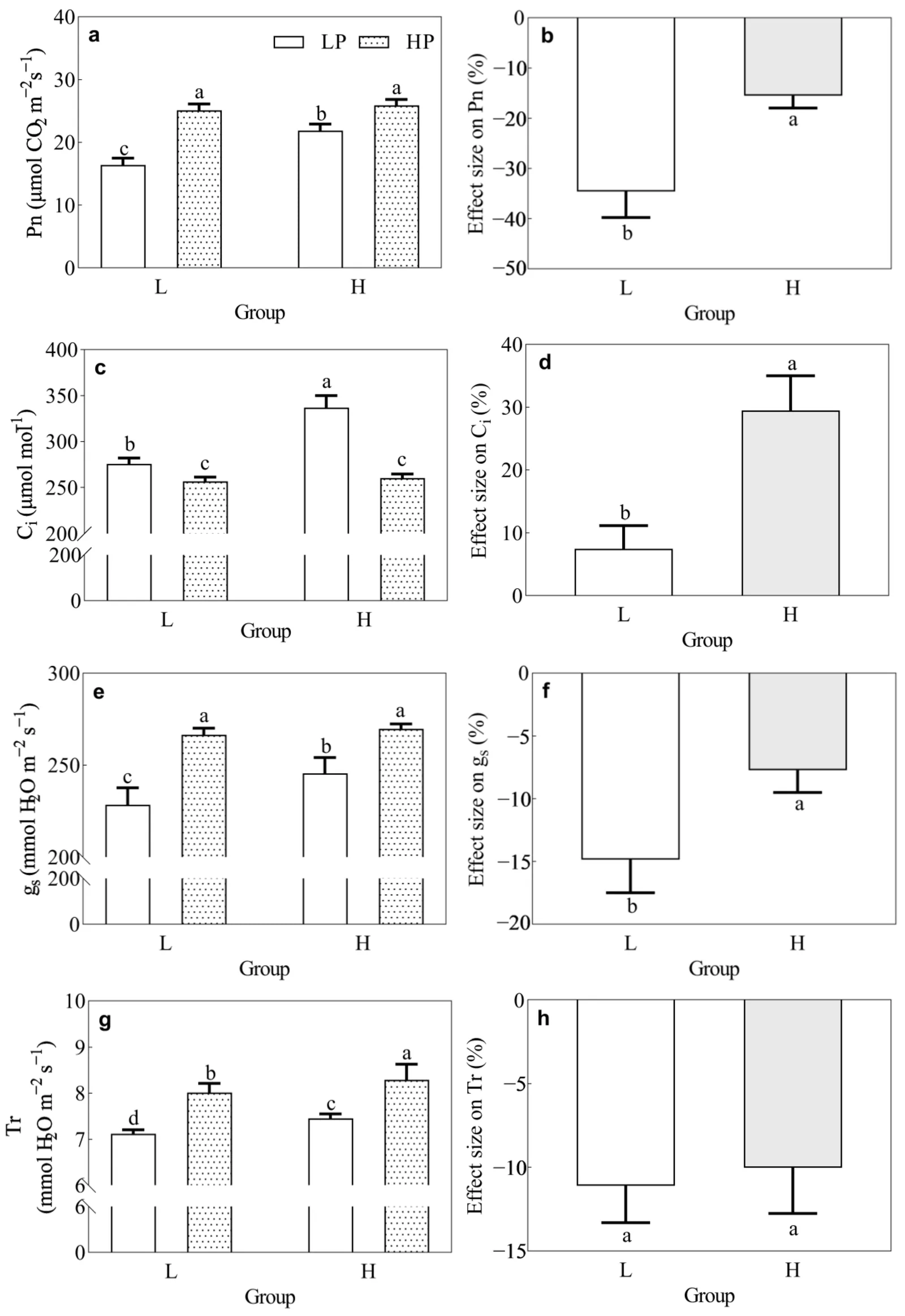
Comparative analysis of leaf gas exchange parameters and response plasticity in the high-Pn (H) and low-Pn (L) peanut accessions under varying phosphorus availability. (**a**,**c**,**e**,**h**) Absolute values of net photosynthetic rate (Pn) (**a**), intercellular CO_2_ concentration (C_i_) (**c**), stomatal conductance (g_s_) (**e**), and transpiration rate (Tr) (**h**) under HP control and LP stress conditions. (**b**,**d**,**f**,**h**) LP-response effect size (%) for Pn (**b**), C_i_ (**d**), g_s_ (**f**), and Tr (**h**). Data are presented as means ± SE (*n* = 3). For absolute values (**a**,**c**,**e**,**g**), a two-way ANOVA was performed to evaluate the main effects of phosphorus treatment (P), accession group (G), and their interaction (P × G) (Table S1); different lowercase letters indicate significant differences among all treatment combinations. For response effect sizes (**b**,**d**,**f**,**h**), data were analyzed using a one-way ANOVA to compare the groups. Different lowercase letters above the bars indicate significant differences among means at *p* < 0.05 according to Fisher’s least significant difference (LSD) test.

Compared with the HP control, LP stress significantly inhibited the Pn across all studied accessions, with reductions of 34.67% and 15.51% observed in the L and H groups, respectively (Figure 1a). Analysis of the LP-response effect size further confirmed that the Pn of the L group was more severely inhibited than that of the H group (Figure 1b). Concurrently, the intercellular CO_2_ concentrations (C_i_s) in both the L and H groups were significantly increased under low-P stress relative to the HP control, with increments of 7.44% in the L group and 29.43% in the H group (Figure 1c). Notably, the effect size of C_i_ was significantly greater in the H group than that of the L group, reflecting a more pronounced adaptive plasticity in response to low-P stress (Figure 1d). Under LP conditions, the stomatal conductance (g_s_) of the L and H groups decreased by 14.87% and 7.74%, respectively, compared with their corresponding HP controls (Figure 1f). This indicates that the L group exhibited a heightened stomatal sensitivity to P deficiency, with a reduction in g_s_ nearly twofold greater than that observed in the H group. A consistent downward trend under LP stress was also observed for transpiration rate (Tr); however, in contrast to the stomatal response, there was no significant difference in the plasticity of Tr between the L and H groups (Figure 1h). In summary, these findings highlight that under P-limited conditions, the H group maintains a distinct physiological advantage in leaf gas exchange; by coupling higher absolute Pn and C_i_ values with less restrictive stomatal control (g_s_), this functional cohort sustains a superior and more resilient carbon assimilation capacity relative to the highly sensitive L group (Figure 1).

### 3.3. Photosystem Activity

**Figure 2:**
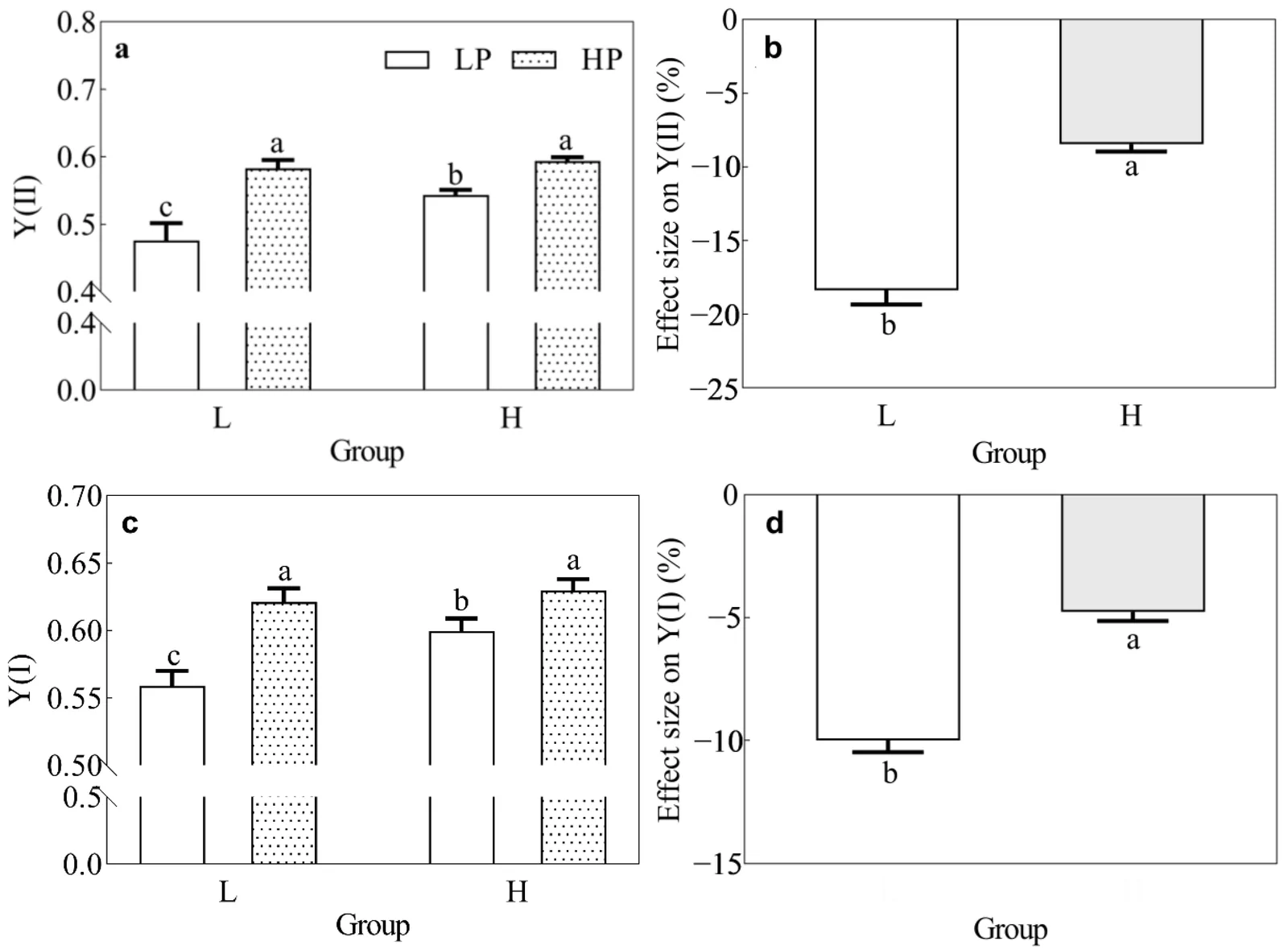
Comparative analysis of photosystem effective quantum yields and response plasticity in high-Pn (H) and low-Pn (L) peanut accessions under varying phosphorus availability. (**a**,**c**) Absolute values of the effective quantum yield of PSII [Y(II)] (**a**) and PSI [Y(I)] (**c**) under HP control and LP stress conditions. (**b**,**d**) LP-response effect size (%) representing the relative changes in Y(II) (**b**) and Y(I) (**d**) induced by LP stress. Data are presented as means ± SE (*n* = 3). For absolute values (**a**,**c**), a two-way ANOVA was performed to evaluate the main effects of phosphorus treatment (P), accession group (G), and their interaction (P × G) (Table S1); different lowercase letters indicate significant differences among all treatment combinations. For response effect sizes (**b**,**d**), data were analyzed using a one-way ANOVA to compare the groups. Different lowercase letters above the bars indicate significant differences among means at *p* < 0.05 according to Fisher’s least significant difference (LSD) test.

Compared with the HP control, the effective quantum yields of both PSI and PSII, Y(I) and Y(II), decreased significantly under LP stress across all studied peanut accessions, confirming the occurrence of stress-induced photoinhibition (Figure 2). Notably, the H group exhibited a significantly less pronounced degree of photoinhibition than the L group (Figure 2b,d). Furthermore, across all investigated accessions, the magnitude of functional suppression in PSI was substantially lower than that in PSII, indicating that PSI was preferentially safeguarded relative to PSII under low-P stress (Figure 2).

### 3.4. Photosystem Protection Mechanisms

**Figure 3:**
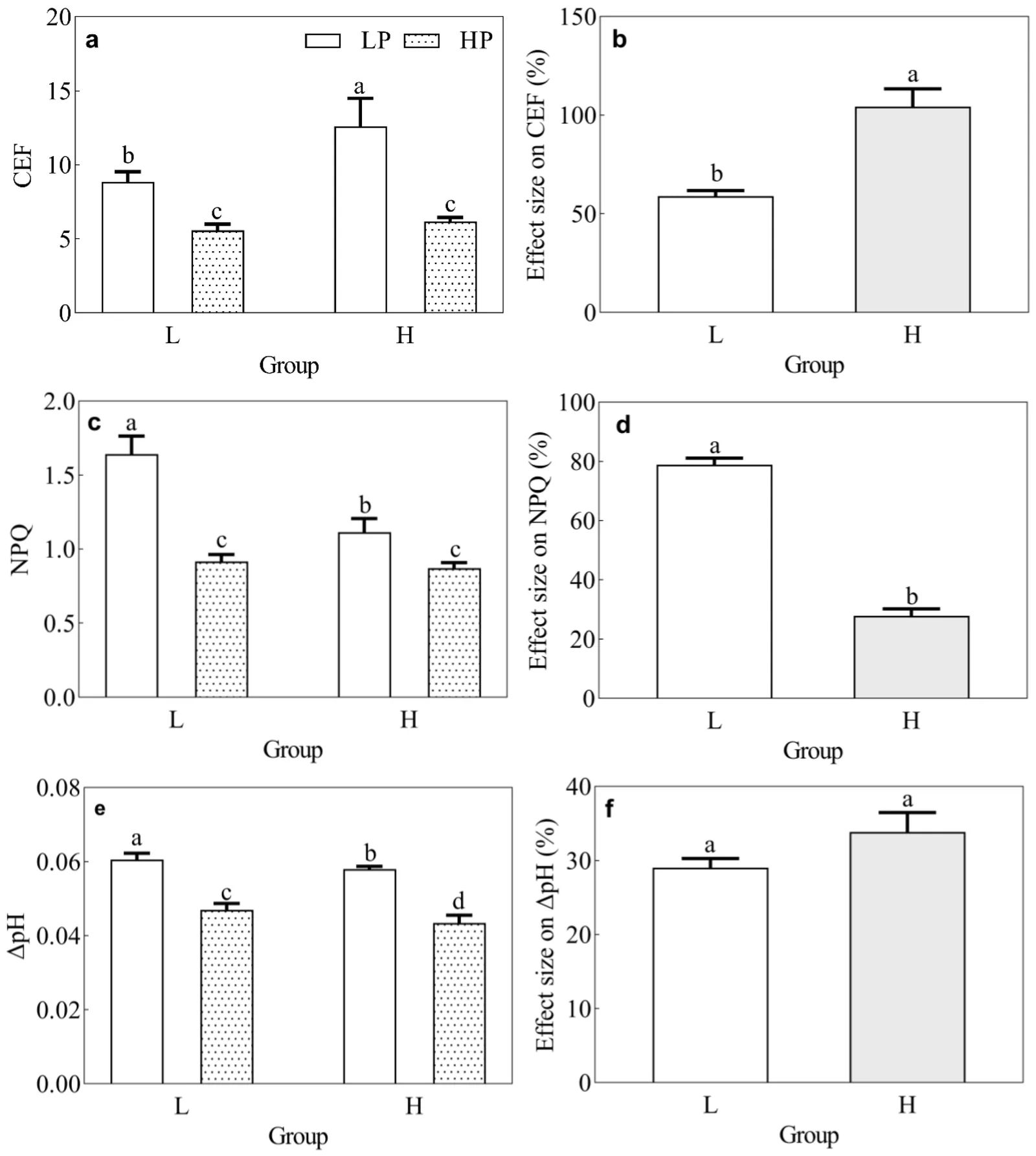
Comparative analysis of photoprotective mechanisms and response plasticity in high-Pn (H) and low-Pn (L) peanut accessions under varying phosphorus availability. (**a**,**c**,**e**) Absolute values of cyclic electron flow (CEF), non-photochemical quenching (NPQ), and trans-thylakoid proton gradient (ΔpH) under HP control and LP stress conditions. (**b**,**d**,**f**) LP-response effect size (%) representing the relative changes in CEF, NPQ, and ΔpH induced by LP stress. Data are presented as means ± SE (*n* = 3). For absolute values (**a**,**c**,**e**), a two-way ANOVA was performed to evaluate the main effects of phosphorus treatment (P), accession group (G), and their interaction (P × G) (Table S1); different lowercase letters indicate significant differences among all treatment combinations. For response effect sizes (**b**,**d**,**f**), data were analyzed using a one-way ANOVA to compare the groups. Different lowercase letters above the bars indicate significant differences among means at *p* < 0.05 according to Fisher’s least significant difference (LSD) test.

Compared with the HP control, cyclic electron flow was significantly upregulated across all studied peanut accessions under LP conditions (Figure 3a). The LP-induced response effect size of CEF in the L and H groups reached 58.76% and 104.5%, respectively, with the upregulation in the H group being significantly more pronounced than that in the L group (Figure 3b). Compared with the HP control, the non-photochemical quenching (NPQ) coefficient increased for all accessions under the LP treatment (Figure 3c). Interestingly, the P-response effect size of NPQ reached 79.00% in the L group but was only 27.96% in the H group, demonstrating that the L group exhibited a markedly higher capacity for NPQ induction relative to the H group (Figure 3d). Furthermore, the trans-thylakoid proton gradient (ΔpH) was significantly increased in all accessions under LP conditions (Figure 3e). Although the absolute magnitude of ΔpH in the H group was significantly greater than that in the L group under low-P stress, no significant difference was observed in their LP-induced response effect sizes on ΔpH. (Figure 3f).

### 3.5. Oxidative Stress Cascades and Antioxidant Defense Plasticity

**Figure 4:**
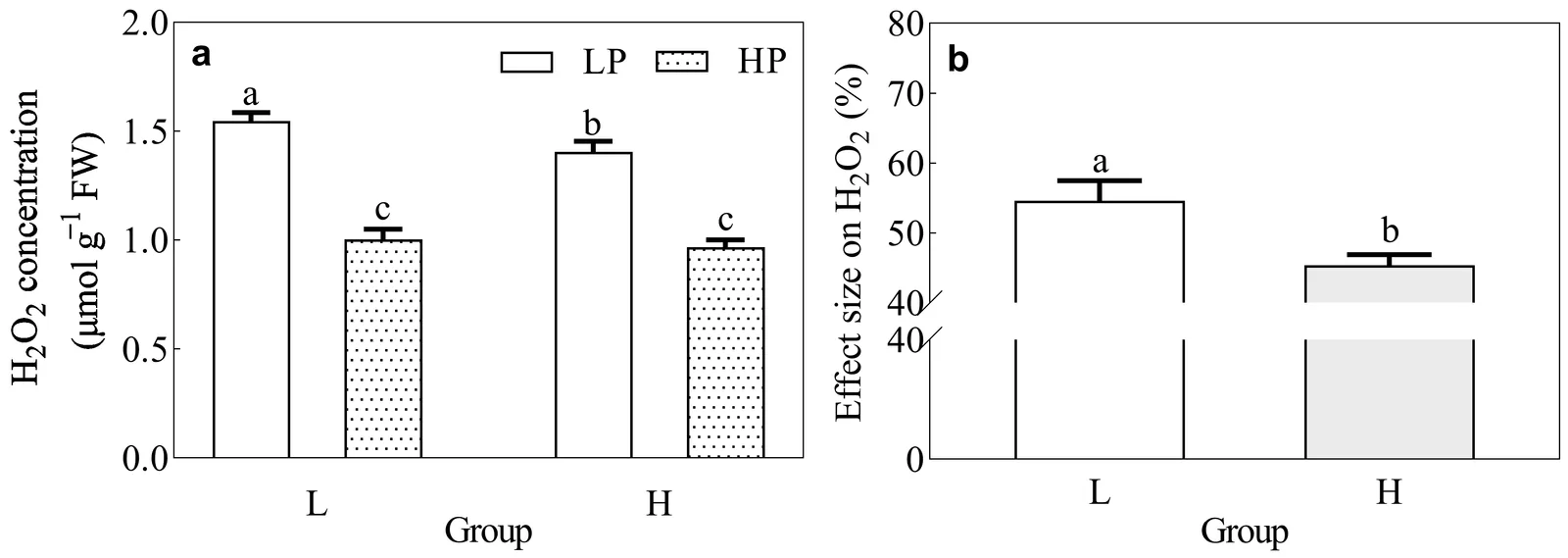

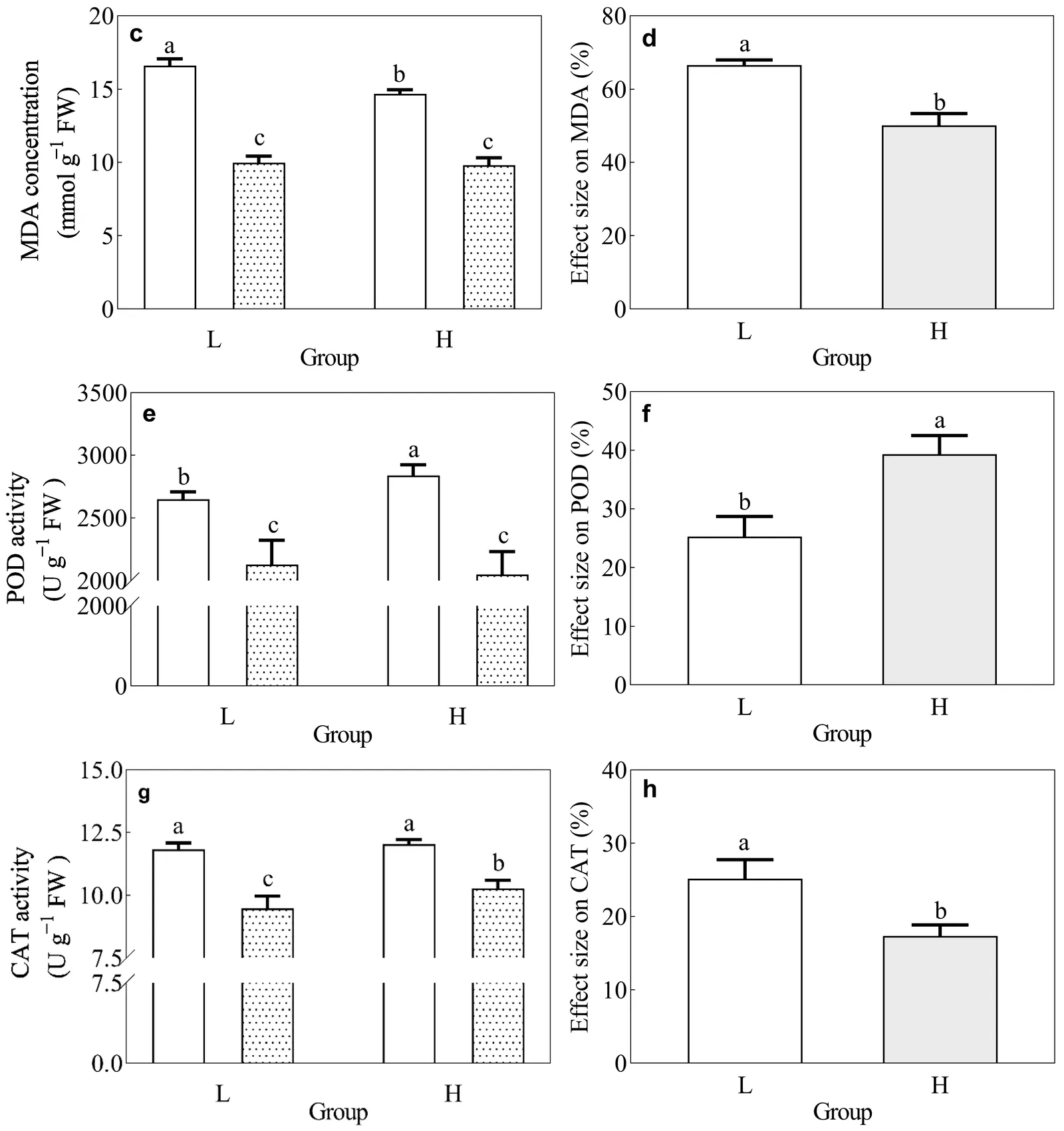
Comparative analysis of oxidative stress biomarkers and antioxidant defense plasticity in high-Pn (H) and low-Pn (L) peanut accessions under varying phosphorus availability. (**a**,**c**,**e**,**g**) Absolute values of hydrogen peroxide (H_2_O_2_) concentration, malondialdehyde (MDA), peroxidase (POD) activity, and catalase (CAT) activity under HP control and LP stress conditions. (**b**,**d**,**f**,**h**) LP-response effect size (%) representing the relative changes in H_2_O_2_, MDA, POD, and CAT induced by LP stress. Data are presented as means ± SE (n = 3). For absolute values (**a**,**c**,**e**,**g**), a two-way ANOVA was performed to evaluate the main effects of phosphorus treatment (P), accession group (G), and their interaction (P × G) (Table S1); different lowercase letters indicate significant differences among all treatment combinations. For response effect sizes (**b**,**d**,**f,h**), data were analyzed using a one-way ANOVA to compare the groups. The legend outlined in panel a is applicable to panels (**a**,**c**,**e**,**g**). Different lowercase letters above the bars indicate significant differences among means at *p* < 0.05 according to Fisher’s least significant difference (LSD) test.

Compared with the HP control, H_2_O_2_ levels in the leaves of both the L and H groups surged remarkably under low-P conditions (Figure 4a). Under HP control, no significant difference in leaf H_2_O_2_ concentration was observed between the L and H groups (Figure 4a). However, under low-P conditions, the H_2_O_2_ concentration in the H group was significantly lower than that in the L group (Figure 4a). Correspondingly, the LP-response effect size for the H_2_O_2_ concentration reached 54.58% in the L group, whereas it was restricted to 45.35% in the H group (Figure 4b). As a critical indicator of membrane lipid peroxidation, the MDA concentration in the leaves of both groups exhibited a sharp elevation under low-P conditions relative to the HP control (Figure 4c). Notably, the LP-induced MDA concentration in the H group was maintained at a significantly lower level compared to the L group (Figure 4c). The specific LP-response effect size on MDA concentration was 66.53% for the L group, while it was 50.14% for the H group (Figure 4d).

Under low-P conditions, the enzymatic antioxidant system was strongly triggered, as evidenced by the synchronized activation of both POD and CAT in all tested plants (Figure 4e,g). Under low-P conditions, the POD activity in the L group was lower than that in the H group; correspondingly, the LP-response effect size for POD was 25.27% in the L group, whereas it reached a substantially higher value of 39.30% in the H group (Figure 4e,f).

Interestingly, no significant difference in absolute CAT activity was detected between the L and H groups under the LP challenge, yet the LP-response effect size of CAT in the L group was significantly greater than that in the H group (Figure 4g,h). This distinct response pattern is heavily attributed to the higher constitutive baseline of CAT activity in the H group under control conditions (Figure 4g), which mitigated the necessity for extreme physiological fluctuations under low-P stress.

### 3.6. Comprehensive Multivariate Assessment

**Figure 5:**
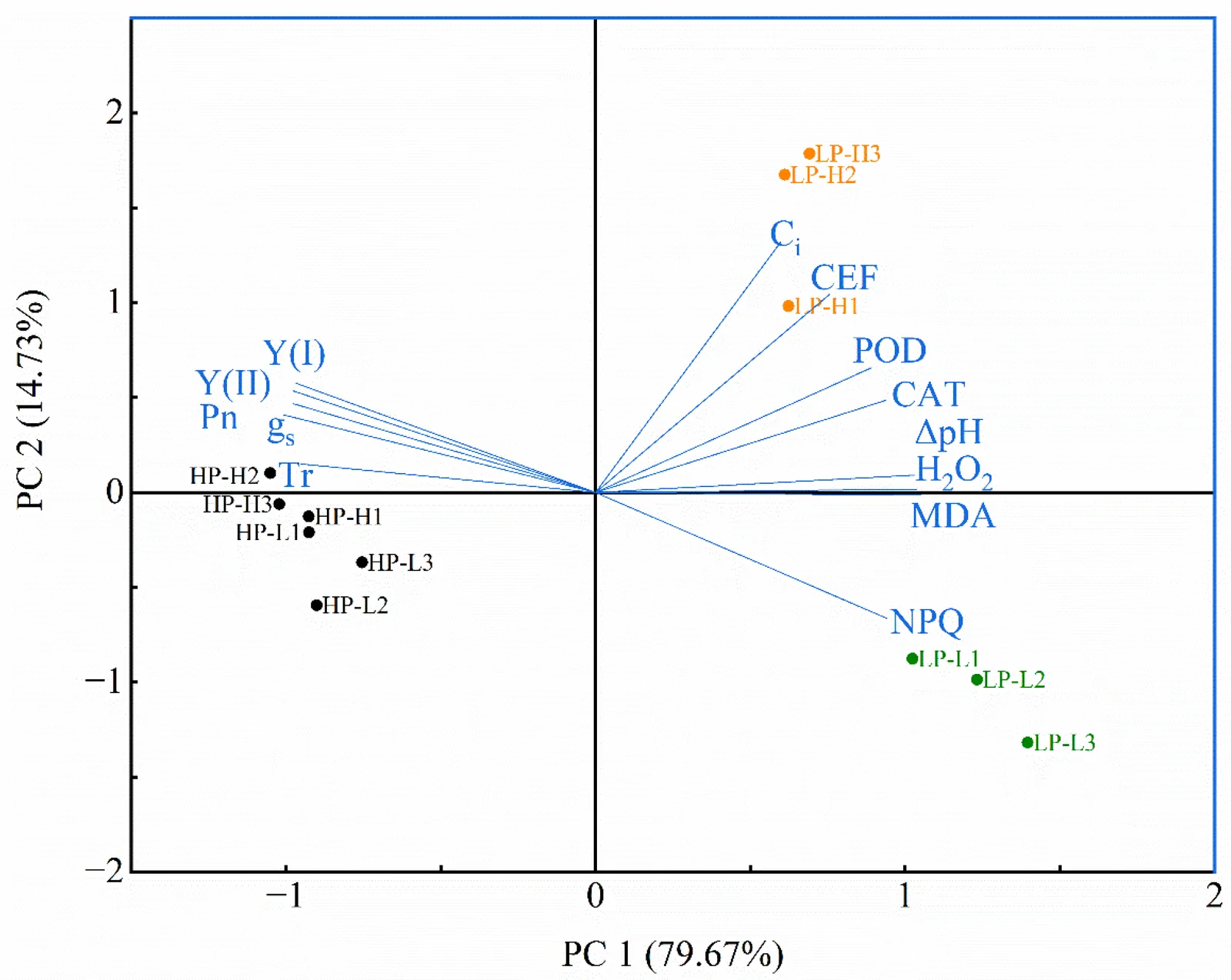
Principal component analysis (PCA) of photosynthetic and chlorophyll fluorescence parameters in peanut leaves under varying phosphorus availability. H1–H3 and L1–L3 represent the three high-Pn and three low-Pn peanut accessions, respectively. The PCA biplot is based on the absolute values of all measured traits under HP control and LP stress conditions. The vectors represent the loading of each trait on the first two principal components. Pn, net photosynthetic rate; C_i_, intercellular CO_2_ concentration; g_s_, stomatal conductance; Tr, transpiration rate; Y(II), the effective quantum yield of PSII; Y(I), effective quantum yield of PSI; CEF, cyclic electron flow; NPQ, non-photochemical quenching; ΔpH, trans-thylakoid proton gradient; H_2_O_2_, hydrogen peroxide; MDA, malondialdehyde; POD, peroxidase activity; CAT, catalase activity.

Principal component analysis (PCA) was performed to evaluate the overall physiological variations across accessions and treatments, with PC1 and PC2 explaining 79.67% and 14.73% of the total variance, respectively (totaling 94.40%) (Figure 5). The separation along the PC1 axis was primarily driven by Pn, g_s_, Tr, Y(I), Y(II), CEF, NPQ, ΔpH, POD, CAT, MDA, and H_2_O_2_ (Figure 5). Under HP conditions, all accessions clustered in the negative direction of PC1, correlating strongly with high Y(I), Y(II), g_s_, Tr, and Pn (Figure 5). Conversely, the LP-treated accessions shifted toward the positive direction of PC1, associating with increased C_i_, CEF, POD, CAT, H_2_O_2_, MDA, NPQ, and ΔpH. Notably, a clear genotypic divergence was observed along the PC2 axis under LP stress: the H group clustered toward the C_i_, CEF, CAT, and POD directions, whereas the L accessions dispersed along the NPQ (Figure 5).

**Figure 6:**
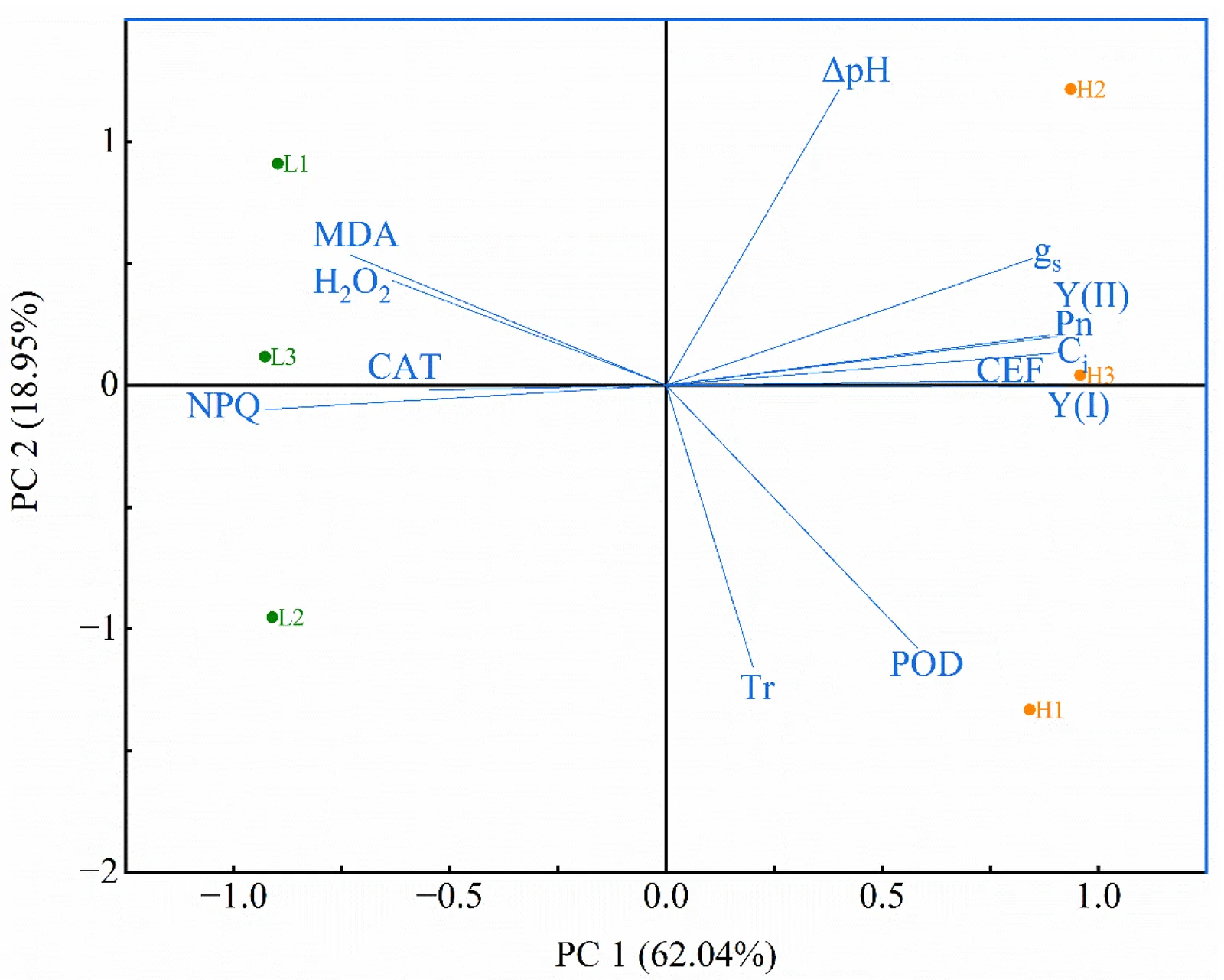
Principal component analysis (PCA) of the LP-response effect size for photosynthetic and fluorescence traits. The analysis was performed using the LP-response effect size of each parameter to evaluate accession differentiation in P stress adaptation. H1–H3 (high-Pn group) and L1–L3 (low-Pn group) are clustered based on their physiological plasticity. The vectors represent the loading of each trait on the first two principal components. Pn, net photosynthetic rate; C_i_, intercellular CO_2_ concentration; g_s_, stomatal conductance; Tr, transpiration rate; Y(II), the effective quantum yield of PSII; Y(I), effective quantum yield of PSI; CEF, cyclic electron flow; NPQ, non-photochemical quenching; ΔpH, trans-thylakoid proton gradient; H_2_O_2_, hydrogen peroxide; MDA, malondialdehyde; POD, peroxidase activity; CAT, catalase activity.

Further PCA of the response plasticity of the photosynthetic and fluorescence parameters in different peanut accessions showed that PC1 and PC2 accounted for 62.04% and 18.95% of the variance, respectively (totaling 80.99%) (Figure 6). The L and H groups were distinguished on PC1 and were clearly distinguished along the PC1 axis, with L accessions associating with the NPQ-mediated photoprotection mechanism, whereas H accessions were characterized by the CEF-mediated mechanism (Figure 6).

## 4. Discussion

### 4.1. Stability of Carbon Assimilation and Pn Plasticity: The Phenotypic Basis of Endogenous Resistance

Under phosphorus deficiency, the concomitant decline in Pn and elevation in C_i_ (Figure 1a,c) confirmed that non-stomatal limitations predominate in governing the overall photosynthetic inhibition across peanut accessions Farquhar and Sharkey [37]. Mechanistically, the low-P stress suppressed sink demand and biomass accumulation, which induced a strong feedback inhibition on Calvin–Benson cycle enzymes [10,14,28]. Furthermore, the P limitation restricted RuBP regeneration and thylakoid ATP synthesis, thereby directly impairing dark-reaction efficiency and imposing a primary biophysical bottleneck on light utilization [15,38].

Beyond these generalized non-stomatal constraints, our multivariate analyses identified stomatal behavior as the key determinant of genotypic divergence in photosynthetic resilience. Low-Pn accessions displayed a significantly tighter stomatal closure and diminished stomatal g_s_ compared to high-Pn accessions under low P (Figure 1, Figure 5 and Figure 6). This divergent stomatal sensitivity directly dictates internal CO_2_ availability, thereby modulating the balance between energy production and consumption. The more pronounced stomatal shutdown in low-Pn accessions inevitably exacerbates the imbalance between the light-harvesting and dark carbon assimilation reactions, leading to immediate, hazardous accumulation of excess excitation energy. To safeguard the photosynthetic machinery against low-P-induced photo-oxidative damage and irreversible photoinhibition under such severe energy-straining conditions, these peanut accessions must dynamically deploy an array of robust endogenous photoprotective mechanisms.

### 4.2. Plasticity of Photoprotective Mechanisms: CEF-Driven Energy Partitioning Strategies

Our results indicate that LP stress induces significant photoinhibition within the photosynthetic apparatus of peanut, with the magnitude of functional impairment being substantially more pronounced in PSII than in PSI (Figure 2). This represents a ubiquitously recognized physiological response in plants subjected to P deficiency [39,40], a pattern consistently corroborated by prior investigations on peanut under P-limited regimes [10,14,28]. The differential sensitivity of PSI and PSII to photoinhibition is primarily dictated by their divergent strategies for managing excitation energy and excess electron flow [25]. Crucially, compared with the rapid turnover and repair cycle of the PSII reaction center core protein (D1), the structural remediation of a damaged PSI complex is exceptionally difficult, metabolically expensive, and time-consuming [25,41]. Consequently, the ΔpH-dependent photosynthesis-control and the CEF-mediated photoprotective mechanism operate in close coordination, functioning as a strategically integrated barrier dedicated to the robust safeguarding of PSI [41,42,43]. Although this regulatory asymmetric sacrifice inevitably subjects PSII to more severe photoinhibition, it serves as an indispensable physiological buffer that shields the highly vulnerable PSI from irreversible photo-oxidative destruction [44]. These findings underscore that PSI-prioritized photoprotection under LP stress represents an evolutionary trade-off in energy allocation, the functional efficacy and plastic capacity of which vary significantly across different peanut accessions, directly underpinning the observed genotypic divergence in Pn resilience.

**Figure 7:**
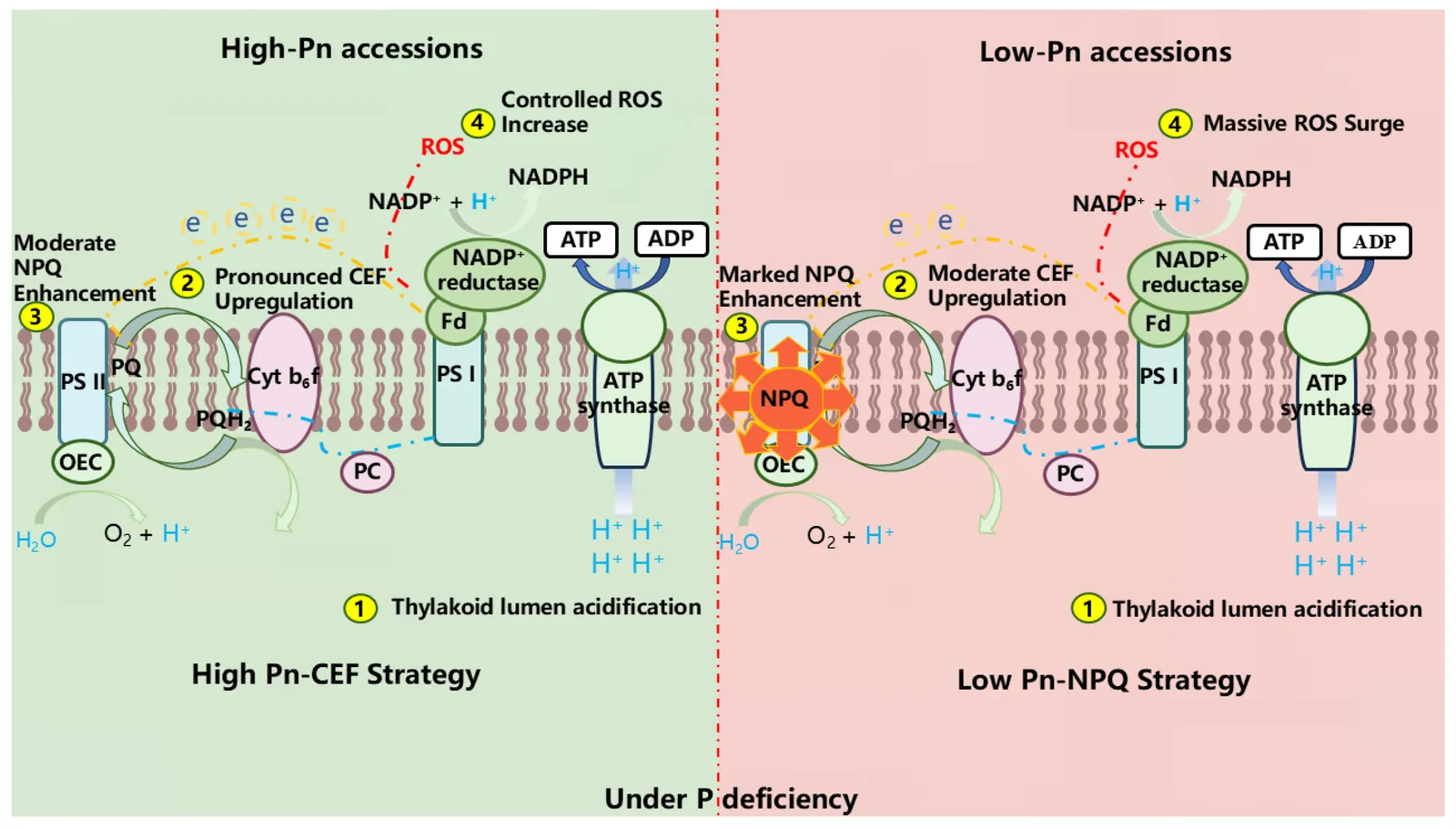
Proposed physiological model for the trade-off between CEF-mediated photoprotection and NPQ-based energy dissipation under P deficiency.

Our results indicate that CEF is significantly upregulated across all studied peanut accessions under LP stress (Figure 3a), serving as an indispensable compensatory response to mitigate LP-induced photoinhibition [10,14]. The CEF around PSI operates as a vital photoprotective mechanism, primarily by modulating the ATP/NADPH balance, safeguarding PSI, and sustaining the ΔpH under LP stress [17,45]. Crucially, given the extreme vulnerability of PSI to electron over-accumulation and its exceptionally sluggish structural repair capacity compared with PSII, CEF-mediated energy routing is widely recognized as the predominant line of defense shielding PSI from irreversible photo-oxidative degradation [46]. Beyond direct photoprotection, CEF around PSI plays a pivotal role in modulating linear electron flow (LEF) via the retrograde feedback downregulation of the cytochrome b_6_f complex [25,43,47]. Under LP stress, the thylakoid lumen of peanuts with both the low- and high-Pn underwent significant acidification, although no statistically significant divergence was detected between the two groups regarding their response plasticity (Figure 3e,f). The P-deficiency-induced increase in ΔpH, which is sustained by CEF-PSI to drive ATP synthesis via proton translocation, downregulates the activity of the cytb_6_f complex and subsequently alleviates the excessive electron transfer from PSII to PSI [10,29,48,49,50]. In the present study, the high-Pn accessions exhibit greater plasticity in CEF than low-Pn accessions, which likely represents the fundamental mechanism underlying the sustained photosystem activity observed in high-Pn accessions under LP stress (Figure 3a,b). Interestingly, peanut accessions with low Pn exhibited higher NPQ values and greater NPQ plasticity under LP conditions (Figure 3c,d), suggesting a heavy reliance on thermal dissipation to mitigate excess excitation energy. Mechanistically, NPQ is strictly dependent on the accumulation of protons within the thylakoid lumen, which alters the protonation state of light-harvesting complexes to trigger its energy-dependent (qE) quenching component [51,52]. Integrating the absolute values of the ΔpH (Figure 3e), its plasticity (Figure 3f), and the multi-trait PCA trajectories (Figure 5 and Figure 6), the establishment of a robust ΔpH is equally indispensable for both high- and low-Pn accessions in coordinating photoprotective responses. The ΔpH sustained by CEF under stressful conditions provides the mechanistic basis for triggering both photosynthetic control and NPQ-mediated photoprotection [48,53,54]. The CEF-NPQ coupling regulatory mechanism promotes crop growth under intense light conditions [55]. Collectively, these findings reveal that contrasting peanut genotypes deploy highly divergent photoprotective strategies under LP stress. Specifically, the high-Pn group prioritizes CEF, whereas the low-Pn group relies predominantly on NPQ-mediated thermal dissipation (Figure 7). Nevertheless, the generation of a robust trans-thylakoid ΔpH stands as a shared and non-negotiable physiological prerequisite for both functional groups (Figure 7). This core bioenergetic foundation is essential to effectively alleviate excitation pressure and ultimately preserve PSI stability.

### 4.3. Synergy Between Photoprotection and Antioxidant Systems Underpins Low-Phosphorus Resilience

Our results indicate that, under low-P conditions, peanuts across different groups experienced varying degrees of P-deficiency-induced oxidative stress (Figure 4a), which subsequently led to oxidative damage to cell membranes and triggered severe membrane lipid peroxidation (Figure 4b). Mechanistically, low-P stress restricts carbon assimilation, thereby entrapping excess electrons within the chloroplast photosynthetic electron transport chain of peanut leaves and causing a profound surge in intracellular ROS [14,18]. Strikingly, this reactive oxygen burst was effectively suppressed in the H group through a highly coordinated synchronization: namely, a superior low-P response plasticity of POD activity coupled with a robust constitutive baseline of CAT activity (Figure 4). As a high-turnover H_2_O_2_-degrading enzyme, CAT operates in an energy-efficient manner without consuming valuable intracellular reducing equivalents; however, while it exhibits strict specificity toward H_2_O_2_, it remains less effective against bulky organic peroxides [56]. Conversely, functioning as a versatile stress-responsive enzyme, POD is heavily induced by adverse environmental coordinates, which strategically reinforces its catalytic efficiency to intercept and quench the intermediate peroxyl radicals and organic oxidants [56,57]. Consequently, this spatial and substrate-specific coordination between CAT and POD constructs a robust, energy-efficient defense network, which effectively neutralizes multi-form ROS, encapsulates the fragile chloroplast thylakoid membrane from catastrophic photo-oxidative disruption, and ultimately bolsters the photosynthetic resilience of peanuts under low-P constraints.

To evaluate the consistency and genotypic variation in photoprotective strategies among peanut accessions under LP conditions, the PCAs were performed based on both absolute core photosynthetic traits (Figure 5) and their corresponding low-P response plasticity indices (Figure 6). In the multivariate space of absolute physiological traits, the first two principal components collectively explained 94.40% of the total variance. Specifically, PC1 (79.67%) primarily captured the variation between HP control and LP treatments, whereas PC2 (14.73%) successfully differentiated between high- and low-Pn accessions under the LP treatment (Figure 5). In the PCA of P response plasticity across key physiological traits, PC1 and PC2 cumulatively explained 80.99% of the total variation. PC1 alone accounted for 62.04%, consistently segregating the high- and low-Pn peanut accessions (Figure 6). Under HP control, photoprotective mechanisms remain largely quiescent, exerting a negligible impact on active carbon assimilation. However, as ΔpH-mediated photosynthetic control, NPQ, and CEF collectively constitute a robust photoprotective network for PSI [48], a significant strategic trade-off emerges among peanut accessions with divergent tolerances to P-deficiency-induced photosynthetic impairments. Under LP stress, a clear strategic divergence emerges: high-Pn accessions prioritize the high-Pn-CEF strategy, whereas low-Pn accessions shift toward the low-Pn-NPQ strategy; the vector of highly plastic POD closely coordinated with CEF, whereas the CAT response vector aligned with NPQ. Building upon these mechanistic insights, CEF-mediated photoprotective traits provide non-destructive, rapid screening biomarkers for evaluating large peanut germplasm panels under low-P stress. Stacking these above-ground functional traits with below-ground nutrient-acquisition capacities will significantly accelerate the breeding of phosphorus-efficient cultivars. While this study elucidates leaf-level photoprotective trade-offs in six peanut accessions under P deficiency, several limitations remain, including the lack of root architectural traits, subcellular ROS localization, and molecular or inhibitor assays targeting key CEF components (PGR5/PGRL1/NDH). Future research incorporating larger accession panels, root-system dynamics, and functional molecular analyses will be essential to definitively validate this proposed physiological model.

## 5. Conclusions

Under LP conditions, peanut accessions exhibited substantial variation in photosynthetic response plasticity, with non-stomatal factors representing the universal determinant constraining absolute carbon assimilation across all accessions. Furthermore, a significant genotypic divergence exists in the response plasticity of photosystem effective quantum yields. Multivariate analysis clarified a distinct strategic trade-off between their adaptive pathways. The high-Pn accessions relied upon the active “CEF-POD” (high-Pn-CEF strategy) to dynamically shunt excess electrons and reinforce ROS scavenging, while leveraging a superior constitutive baseline of peroxisomal CAT to clear H_2_O_2_ in an energy-efficient manner. Conversely, the low-Pn accessions failed to sustain this eco-energetic balance and instead shifted toward the passive “NPQ-CAT” (low-Pn-NPQ strategy) as a systemic safety valve under severe oxidative overload. This study elucidated the plausible mechanistic trade-off between CEF around PSI and NPQ-based thermal dissipation across contrasting peanut accessions, offering novel insights into the physiological adaptation of peanuts to P deficiency while providing a scientific framework for the development of targeted functional foliar fertilizers for peanuts.

## Figures and Tables

**Table 1:** The classification of the high-Pn (H) and low-Pn (L) groups was based on the photosynthetic performance (specifically net CO_2_ assimilation rate) of selected peanut accessions under low-P stress. A composite substrate consisting of Liaoning brown soil and washed, sun-dried river sand in a 1:1 (*v*/*v*) ratio was used in this study. Data are presented as means ± SE (*n* = 3). Different lowercase letters within the same column indicate significant differences among means according to Fisher’s least significant difference (LSD) test (*p* < 0.001).

Group	Accession	Individual Accession CO_2_ Assimilation Rate (μmol CO_2_ m^−2^ s^−1^)	Group Mean CO_2_ Assimilation Rate (μmol CO_2_ m^−2^ s^−1^)
L	ICG928	17.70 ± 0.35 c	16.40 ± 0.36 b
	ICG1101	15.54 ± 0.28 d	
	ICG1448	15.95 ± 0.29 d	
H	ICG7969	22.83 ± 0.31 a	21.88 ± 0.35 a
	ICG9418	22.19 ± 0.21 a	
	ICG13603	20.61 ± 0.16 b	

## Data Availability

The original contributions presented in the study are included in the article/Supplementary Material; further inquiries can be directed to the corresponding author.

## Supplementary Materials

The following supporting information can be downloaded at https://www.mdpi.com/article/10.3390/antiox15081002/s1, Table S1: Two-way analysis of variance (ANOVA) showing the main effects of phosphorus (P) levels, accession groups, and their interactions on photosynthetic parameters and antioxidant enzyme activities in peanut leaves.
