# Supplementary material for: Phenotypic Plasticity of Photochemical Traits and Antioxidant Responsiveness Confer Photosynthetic Resilience in Peanut (Arachis hypogaea L.) Under Phosphorus Deficiency: The Pivotal Role of Cyclic Electron Flow
Source: Antioxidants (Basel). 2026 Aug 12;15(8):1002. doi: 10.3390/antiox15081002 (PMC13509911; doi:10.3390/antiox15081002)
Supplement: Supplementary file 1 [file antioxidants-15-01002-s001.zip › antioxidants-4434276-supplementary.pdf]

Supplementary Materials

Phenotypic Plasticity of Photochemical Traits and Antioxidant Responsiveness Confer Photosynthetic Resilience in Peanut (*Arachis hypogaea* L.) Under Phosphorus Deficiency: The Pivotal Role of Cyclic Electron Flow

**Table S1.** Two-way analysis of variance (ANOVA) showing the main effects of phosphorus (P) levels, accession groups, and their interactions on photosynthetic parameters and antioxidant enzyme activities in peanut leaves.

| | **Significance** | | |
|---|---|---|---|
| | **P Level** | **Accession Group** | **Interaction** |
| Pn (μmol $CO_2$ $m^{-2}$ $s^{-1}$) | *** | *** | *** |
| $C_i$ (μmol $mol^{-1}$) | *** | *** | *** |
| $g_s$ (mmol $H_2O$ $m^{-2}$ $s^{-1}$) | *** | *** | ** |
| Tr (mmol $H_2O$ $m^{-2}$ $s^{-1}$) | *** | *** | ns |
| Y(II) | *** | *** | *** |
| Y(I) | *** | *** | *** |
| CEF | *** | *** | *** |
| NPQ | *** | *** | *** |
| ΔpH | *** | *** | ns |
| $H_2O_2$ (μmol $g^{-1}$) | *** | *** | *** |
| MDA (mmol $g^{-1}$) | *** | *** | *** |
| POD (U $g^{-1}$ FW) | *** | ns | ** |
| CAT (U $g^{-1}$ FW) | *** | *** | * |

Note: Values represent the statistical significance from the two-way ANOVA. ns, not significant; *, $P < 0.05$; **, $P < 0.01$; ***, $P < 0.001$, "ns" indicates "not significant". Pn, net photosynthetic rate; $C_i$, intercellular $CO_2$ concentration; $g_s$, stomatal conductance; Tr, transpiration rate; Y(II), the effective quantum yield of PSII; Y(I), effective quantum yield of PSI; CEF, cyclic electron flow; NPQ, non-photochemical quenching; ΔpH, trans-thylakoid proton gradient; $H_2O_2$, hydrogen peroxide; MDA, malondialdehyde; POD, peroxidase activity; CAT, catalase activity.
